# Consent for publication: Why it matters now more than ever?

**DOI:** 10.1002/ski2.4

**Published:** 2020-09-02

**Authors:** S. Ahmed, A.R. Shipman, G. Millington, E.A. Langan, J.R. Ingram

**Affiliations:** ^1^ British Association of Dermatologists London UK; ^2^ Dermatology Portsmouth Hospitals NHS Trust Portsmouth UK; ^3^ Dermatology Norfolk and Norwich University Hospital Norwich UK; ^4^ Department of Dermatology and Venerology University Hospital of Schleswig Holstein Lübeck Germany; ^5^ Dermatological Sciences University of Manchester Manchester UK; ^6^ Dermatology & Wound Healing Cardiff University University Hospital of Wales Cardiff UK

We live in an age where almost everything we do is in the ‘public eye’ and machine learning is constantly collecting data and images from the Internet. While the integration of ‘big data’ into clinical practice may have profoundly positive effects, it nevertheless poses significant challenges in terms of patient privacy and confidentiality.^1^ In keeping with all medical journals, it is our responsibility to ensure that the articles we publish have patient consent for publication, especially for clinical images or case reports.

Earning and retaining the trust of the public and patients is an important principle.^2^ Article 9 in the General Data Protection Regulation specifically discusses patient confidentiality and how information gathered for research must be handled.^3^ The UK Department of Health’s confidentiality code of practice clearly articulates that consent must be obtained and recorded when a patient's identifiable information is used outside the context of care.^4^

Dermatology is one of the most visual medical specialties and photographs are an integral part of research publications. However, there has always been a grey area around identifiable and non‐identifiable photos. For example, a person's foot may not be identifiable, but if a distinctive tattoo is included in the image then it could be identified. Before the dawn of social media, the patient may never have come across their image in medical literature. In today's publishing world, their image may be viewed and recognized in a social media post by family or friends, resulting in the loss of anonymity. Sometimes the rarer the disease, the easier it is to piece together strands of information, meaning that patients featured in case reports may be identifiable even though the researchers believe this to be unlikely. While the chances are slim, this is eminently possible.

Open access articles allow anyone to view or reuse images, which can pose a problem if a patient has given consent for treatment but not for publication or sharing on social media. In the past, photos were used only within the context of the article and were read or viewed solely by the research community or subscribers, but open access publishing changes this entirely.^5^ A patient who views their image out of the original context for which they gave consent may feel that their privacy has been invaded.^6^ It is our duty as custodians of patients' photos and data to protect them in line with statutory obligations.^3^, ^7^

To address the evolving nature of medical publishing, including social media considerations, we have revised our journal policies and now require authors to obtain written consent from their patients for all case reports and all clinical images, whether or not they are identifiable. In line with recommendations from the Committee for Publication Ethics^8^ and following discussions with our editorial board members, we have clarified our instructions to authors as follows.

1. All authors who submit to the journal will need to confirm during the submission process that they have consent for publication of any clinical images (including those that may not be identifiable) and that they have consent for publication on social media.
2. The British Association of Dermatologists consent form is available online^9^ for use in any of our journals: the *British Journal of Dermatology* (*BJD*), *Clinical and Experimental Dermatology* (*CED*) and *Skin Health and Disease* (*SHD*). In order to protect patient privacy, we do not require completed consent forms to be submitted; however, authors should securely store the consent form themselves.
3. Researchers can use their own institutional consent forms so long as these include consent for publication of the patient's images or case report on the Internet, as well as for social media. We require submission of a blank consent form to demonstrate compliance.

We realize that our revised patient consent policy asks a bit more from authors, but as part of being an ethical and responsible publisher, we are taking this extra step to protect patient privacy and to ensure that trust in medical research is maintained. Fully informed patient consent for publication in the *BJD*, *CED* and *SHD* is a key part of our mission statement to improve patient outcomes in skin disease worldwide, and we thank our authors for ensuring that we all continue to put patients first in all of our publishing endeavours.

## CONFLICT OF INTEREST

Shehnaz Ahmed is Head of Publishing at the British Association of Dermatologists; Alexa R. Shipman is Editor in Chief of *CED*; George Millington is Editor in Chief of *SHD*; Ewan A. Langan is Deputy Editor of *SHD*; and John R. Ingram is Editor in Chief of the *BJD*.
